# DNA Damage Response Mechanisms in Head and Neck Cancer: Significant Implications for Therapy and Survival

**DOI:** 10.3390/ijms24032760

**Published:** 2023-02-01

**Authors:** Chara Papalouka, Maria Adamaki, Panagiota Batsaki, Panagiotis Zoumpourlis, Antonis Tsintarakis, Maria Goulielmaki, Sotirios P. Fortis, Constantin N. Baxevanis, Vassilis Zoumpourlis

**Affiliations:** 1 Biomedical Applications Unit, Institute of Chemical Biology, National Hellenic Research Foundation (NHRF), 11635 Athens, Greece; 2 Cancer Immunology and Immunotherapy Center, Saint Savas Cancer Hospital, 11522 Athens, Greece

**Keywords:** head and neck cancer (HNC), head and neck squamous cell carcinoma (HNSCC), genomic instability, DNA damage response, non-homologous end joining (NHEJ), homologous recombination (HR)

## Abstract

Head and neck cancer (HNC) is a term collectively used to describe a heterogeneous group of tumors that arise in the oral cavity, larynx, nasopharynx, oropharynx, and hypopharynx, and represents the sixth most common type of malignancy worldwide. Despite advances in multimodality treatment, the disease has a recurrence rate of around 50%, and the prognosis of metastatic patients remains poor. HNCs are characterized by a high degree of genomic instability, which involves a vicious circle of accumulating DNA damage, defective DNA damage repair (DDR), and replication stress. Nonetheless, the damage that is induced on tumor cells by chemo and radiotherapy relies on defective DDR processes for a successful response to treatment, and may play an important role in the development of novel and more effective therapies. This review summarizes the current knowledge on the genes and proteins that appear to be deregulated in DDR pathways, their implication in HNC pathogenesis, and the rationale behind targeting these genes and pathways for the development of new therapies. We give particular emphasis on the therapeutic targets that have shown promising results at the pre-clinical stage and on those that have so far been associated with a therapeutic advantage in the clinical setting.

## 1. Introduction

Head and neck cancer (HNC) is a term collectively used to describe a heterogeneous group of neoplasms which include cancers of the lip and oral cavity, the sinus and nasal cavity, the pharynx and larynx, the salivary gland and the thyroid gland, and is associated with poor prognosis in advanced stages [1]. The most common type of HNC is squamous cell carcinoma (HNSCC), accounting for approximately 90% of all HNCs, as most subtypes are derived from squamous cells of the mucosal epithelium [1]. In 2017, the Global Burden of Disease (GBD) study estimated that HNC represents 5.3% of all cancers (excluding nonmelanoma skin cancers), with the lip and oral cavity (LOC) cancers being the most frequent, followed by larynx cancers [2,3]. It has also been estimated that approximately 600,000 new HNSCC cases are diagnosed annually throughout the world, with mortality rates reaching as high as 50% [4,5,6].

The onset of HNC depends on an interplay between genetic factors, such as Fanconi anemia (FA), Bloom syndrome and xeroderma pigmentosum, and environmental factors, such as age, geographic region and various lifestyle contributors [7]. A major risk lifestyle factor is tobacco smoking, while smoking electronic cigarettes, despite being considered less harmful than conventional cigarettes, has also been associated with the development of HNSCC due to their high content of substances that can cause oral mucosal lesions [2]. Another major lifestyle risk factor is alcohol consumption, even in individuals who have never been smokers, while combined use of alcohol and tobacco has been shown to have a synergistic effect on tumor development [2,8,9]. Notably, a significant association, albeit to a lesser extent compared to smoking and alcohol consumption, has also been observed between Human papillomavirus (HPV) infection and the development of various HNCs, especially oropharyngeal (OPC), nasopharyngeal (NPC), oral cavity (OC), and larynx cancers [6]. To date, thirteen types of HPV have been characterized as carcinogenic by the International Agency for Research on Cancer, with the HPV16 subtype being regarded as the most potent contributor to the development of HNC [2,10].

The heterogeneity and diversity of anatomical sites in HNC make its treatment a challenging task. Current approaches include a combination of surgery, radiotherapy, and chemotherapy [11,12]. Radiotherapy (RT) combined with platinum compounds such as cisplatin, with or without 5-fluorouracil, has been the standard of care for many years but has, however, been associated with a modest survival advantage and significant treatment-related mortality [13,14]. More recent chemotherapeutics include taxols and the anti-epidermal growth factor receptor (anti-EGFR) antibody cetuximab, which, despite having been specifically approved for recurrent and metastatic HNC, has also been associated with an improvement in response rates and increased survival in locoregional HNSCC [15,16]. Of note, cancer immunotherapy has emerged in recent years as an additional treatment approach to HNCs, especially in the form of immune checkpoint inhibitors (ICIs), based on the rationale that these monoclonal antibodies have the ability to inhibit immune checkpoint receptors and, therefore, to prevent the inactivation of T-cell function and subsequent cancer immune escape [17]. In this regard, the ICIs nivolumab and pembrolizumab, which specifically bind to immune checkpoint receptor programmed cell death protein 1 (PD-1), have been approved for patients with recurrent or metastatic disease previously treated with platinum chemotherapy, conferring a survival benefit in 20–30% of patients [16,18,19]. However, despite advances in multimodality treatment and promising results from administering therapeutic combinations in clinical trials, relapse rates remain very high, and 5-year survival is still estimated at 66% [19]. This undoubtedly highlights the need for devising new strategies that will target resistant clones and prolong survival.

As with most solid tumors, HNCs are characterized by genomic instability, which may manifest both as chromosomal instability (CIN), in the form of chromosomal rearrangements or changes in chromosomal ploidy, as nucleotide instability (NIN), in the form of gene amplification, deletion, inversion, and base substitution or mutation, or as microsatellite instability (MIN), in the form of loss or addition of oligonucleotide repeats [13,20,21,22]. The mechanism behind these aberrations seems to involve a vicious circle of accumulating DNA damage, defective DNA damage repair (DDR), and replication stress [13,21,23,24]. The latter can induce the collapse of replication forks and the occurrence of DNA double-strand breaks (DSBs), which appear to be a prominent feature of HNCs [21,25,26]. The inability to appropriately repair these DSBs translates to inability to effectively inhibit cell cycle progression for DNA repair or apoptosis, leading to an ever-increasing accumulation of DNA lesions and the creation of a highly unstable genetic environment that is driven by mutagenic stress [13]. Interestingly, a positive correlation has been identified between the number of chromosomal arms bearing allelic loss and tumor grade, neck nodal status, disease recurrence, metastasis, and overall survival in patients with HNC [20,27]. The extent of DNA damage and the efficiency of DNA repair mechanisms are also significantly associated with response to treatment, and may play an important role in the development of novel therapies.

Even though malignant transformation results from a defective DDR mechanism and subsequent development of genomic instability, the induction of DSBs by chemotherapeutic agents and radiotherapy relies on defective cellular DNA repair processes for a successful response to treatment [28]. Overall, DNA repair mechanisms for DSBs fall into two main types: (i) the homology recombination repair (HRR or HR) mechanism, which uses extensive homology and DNA synthesis is facilitated through a template, usually the sister chromatid, in order to regenerate the lost sequences, and (ii) the non-homologous end joining (NHEJ) mechanism, which rejoins DSBs without any homology requirement and is therefore considered an error-prone DNA repair mechanism [28,29,30]. HR occurs largely during the late S and G2 phases, when the double helix on one sister chromatid can provide sequence information to repair the damaged chromatid, while NHEJ occurs predominantly during the G1 phase of the cell cycle, when sister chromatids are unavailable to allow homology-directed repair, and less during S/G2; in other words, NHEJ exhibits decreased fidelity in repairing DSBs compared to HR [30,31]. Even though defects in both mechanisms have been shown to confer a pro-carcinogenic effect, the chemo- and radioresistant properties of certain tumors appear to rely on effective DDR signaling to escape the cell-cycle arrest and apoptotic pathways induced by DSBs [28,32,33,34]. Recent evidence also highlights the existence of tumors with impaired or inactivated DDR pathways, as well as tumors with mutated DNA repair genes that act as tumor suppressors, which may be specifically targeted to improve therapeutic outcomes and patient survival [35,36,37,38].

This review gives an overview of the main DNA repair mechanisms involved in the pathogenesis of HNC, including the different genes and proteins that appear to be aberrantly expressed or mutated in DDR pathways, and how these aberrations are differentially implicated in cancer development and therapeutic resistance. We discuss the rationale behind targeting these genes and pathways for the development of novel therapeutic interventions and give a detailed account of those that have shown promising results in pre-clinical and clinical studies and the potential to confer a therapeutic advantage or survival benefit, either used as monotherapy or in combination with other anti-cancer therapies.

## 2. Genomic Instability in HNC

It has long become evident that genomic instability is implicated in tumor initiation, progression, and survival [21,39], and that it may manifest as one of three main types: chromosomal instability, nucleotide instability (ΝΙΝ), and microsatellite instability [21,22,40]. CΙΝ is the high rate of change in the structure and number of chromosomes in cancer cells in relation to normal cells [21,22]; MIN is the addition or loss of a number of oligonucleotide repeats due to defects in DNA mismatch repair (MMR) [40,41]; whereas NIN is the increased rate of point mutations, including base substitutions, deletions, and insertions, and is believed to occur as a result of base excision repair (BER) and nucleotide excision repair (NER) pathway malfunctioning [40]. Even though heritable germline mutations naturally increase the risk of cancer occurrence, hereditary cancers are also characterized by acquired somatic mutations [41,42]. Additionally, regardless of the type of gene that is affected by the hereditary mutation, i.e., whether the mutation occurs in an oncogene, an onco-suppressor gene, or a gene that is related to DNA repair, the prevailing hypothesis states that genomic instability is present in precancerous lesions and leads to tumor growth by increasing the rate of mutations, thereby supporting the notion of a continuous mutator phenotype (the so-called “mutator hypothesis”) [21,43,44]. Examples of hereditary HNCs include the Gorlin–Goltz and FA syndromes, with the former arising from mutations in the patched tumor suppressor gene (*PTCH*) [45,46] and the latter from mutations in genes implicated in the Fanconi anemia pathway [47], both being characterized by increased genomic instability [48]. On the other hand, in sporadic cancers, the mutations are acquired spontaneously and randomly; however, they also lead to replication stress and increased genomic instability [21,42,49,50].

Replication stress may be induced by both endogenous and exogenous sources. In addition to the overexpression or constitutive activation of oncogenes such as *RAS*, *MYC*, and *EGFR* families, and *cyclin E* [51], replication stress may also arise from replication barriers such as the presence of DNA–RNA hybrids, collisions between replication and transcription complexes [52], altered chromatin compaction and hypo-acetylation, shortened telomeres [53], repetitive sequences, early replicating fragile sites (ERFSs), and common fragile sites (CFSs) [54,55,56]. Additional barriers include DNA lesions and misincorporation of ribonucleotides, secondary DNA structures, and dormant replication origins due to external or internal factors of DNA damage that have not been repaired [54]. Exogenous sources of DNA damage include environmental agents such as chemicals, ultraviolet light, ionizing radiation, toxins, and pollutants, while major sources of endogenous DNA damage include oxidative DNA damage from the presence of reactive oxygen species (ROS), oxygen, and lipid peroxidation products (e.g., MDA-induced DNA damage derived from lipid peroxidation, propano, and etheno adducts), hydrolytic deamination and carbonyl stress, and several estrogen metabolites, among others [57].

Εxposure to carcinogens that are produced by environmental pollution or through lifestyle habits such as tobacco smoking and excessive alcohol consumption, and its association with an increased risk of developing HNCs, have been widely studied [58,59,60,61]. Nitrosamines and polycyclic aromatic hydrocarbons (PAHs) that are formed during tobacco smoking, as well as acetaldehyde production and cytochrome p450 2E1 (CYP2E1) enzyme induction by alcohol consumption, contribute to the formation of persistent DNA adducts, which disrupt the DNA double helix and lead to the formation of hypermutations and chromosomal instability [62,63,64,65,66,67]. Smoking has also been implicated in the dysregulation of antitumor immune mechanisms, and particularly in the impairment of T-cell-mediated immune responses in patients with esophageal squamous cell carcinoma (ESCC) [68]. In addition, betel quid chewing, due to its alkaloid ingredients, has been associated with an increased risk of HNC development [69,70]. Current research suggests that the betel quid ingredient, arecoline, may suppress the expression of *ATM* and *BRCA1* genes, thereby interrupting DDR and repair processes, ultimately leading to carcinogenesis [71].

The consequences of genomic instability in HNCs are reflected in the high heterogeneity of these tumors, which may influence the outcome of targeted therapy and disease prognosis [72]. Specifically, intra-tumoral heterogeneity is associated with poor prognosis in HNC patients [73]. HPV positive (HPV(+)) HNSCCs are significantly different in mutation patterns [29,30], gene expression [74,75,76,77,78], protein abundance profiles, and genome methylation [79,80], as compared to HPV negative (HPV(−)) HNSCCs [81]. For example, most HPV(−) tumors present with mutations in the *p53* gene, which is rarely mutated in HPV(+) tumors, and are also characterized by significantly higher rates of allelic loss and chromosomal alterations [49,82,83,84]. On the other hand, HPV(+) tumors seem to be driven by *E6* and *E7* viral oncogenes, which may be sufficient to deregulate onco-suppressor factors leading to carcinogenesis [49]. Several studies have revealed differences in the expression of cell cycle-related genes in HPV(+) tumors, which may further explain the faster cell proliferation observed in these tumors in comparison with HPV(−) HNCs [49,85,86]. However, the high complexity and heterogeneity in the landscape of HNC are observed in both HPV(−) and HPV(+) HNSCCs.

HPV-associated HNCs present with altered DNA methylation status, with genes in HPV(+) HNSCCs being either hypo or hypermethylated as compared to HPV(−) HNSCCs [87]. However, the majority of HPV(+) HNCs exhibit higher DNA methylation levels than HPV(−) HNCs, and these have been associated with immune responses on virally infected cells and viral invasion processes [88]. Demethylation treatment with 5-azacytidine-generating DSBs seems to be effective in these cancers due to a defective FA-dependent homologous recombination mechanism [89]. The Fanconi anemia pathway is involved in interstrand crosslink (ICL) repair and seems to play an important role in HNSCC prognosis and treatment response [90].

## 3. DDR Mechanisms—A Brief Overview

The cellular response to DNA damage and replication stress is the activation of the DDR mechanisms, which signal the presence of DNA damage and promote its repair, or, in some cases, lead the cell to apoptosis in order to prevent the spread of damage. Apart from NHEJ and HR, several other DNA repair pathways are also included in the DDR armamentarium. These are as follows:

(i) Alternative end joining (A-EJ) is a recently described mechanism that repairs DSBs through a subset of A-EJ pathways and relies on microhomology-mediated repair. The best characterized Alt-EJ pathway is the microhomology end joining (MMEJ) pathway; this requires 2 to 20 nucleotides of homologous sequence and shares the same first steps of DDR with HR, i.e., the repair process begins with end resection and involves many of the factors that contribute to the HR end resection machinery. These mechanisms are considered to be error-prone and highly mutagenic as they are associated with deletions flanking the original DSB, leading to chromosomal rearrangements and genomic instability [91,92,93,94].
(ii) Nucleotide excision repair (NER) is a mechanism that repairs bulky DNA lesions that distort the DNA double helix and are usually caused by external mutagens, such as UV light and chemical carcinogenic substances [91,95]. NER is divided into two sub-pathways: global genome NER (GG-NER), which takes place throughout the genome, independently of transcriptional processes, and transcription-coupled NER, which only occurs in order to repair the damage on the transcribed strand [96,97,98].
(iii) Mismatch repair (MMR) is a mechanism that repairs base–base mismatches, deletions, or insertions predominantly generated during DNA replication and recombination processes or mismatches that escape DNA polymerase proofreading activity. MMR is considered a highly conserved biological pathway and plays an important role in genome maintenance [91,99].
(iv) Base excision repair (BER) is a mechanism that tends to repair DNA damage (small base lesions) that originate from endogenous sources, such as those attributed to ROS, alkylation, deamination, and methylation, and which do not create structural distortions of the DNA double helix [91,100]. BER is initiated by a DNA glycosylase that recognizes and removes the damaged base, leaving an empty space that is further processed by short-patch repair or long-patch repair to complete the repair mechanism. A study in a Pakistani population suggested that the deregulation of genes in the BER pathway may drive HNSCC progression [101].

Below, we discuss proteins related to DDR mechanisms that have been found mutated or aberrantly expressed in HNC cell lines or patients, and their implication in the development, prognosis, and therapy of HNCs.

## 4. DDR Molecules with Evidence of Implication in the Pathogenesis of HNC

### 4.1. DDR Signaling Kinases

In mammalian cells, the main upstream kinases orchestrating the DDR signaling pathway are the ATM (ataxia telangiectasia mutated) and ATR (ATM- and Rad3-related) serine/threonine kinases, while the DNA-PKcs (DNA-dependent protein kinase catalytic subunit) is a DDR sensor regulating a smaller number of targets and plays a primary role in NHEJ [102,103]. However, the upstream molecules of DDR not only play a crucial part in the repair of DNA lesions, but they are also implicated in the regulation of the cell cycle, cell cycle arrest, and apoptosis. For this reason, DDR plays a critical role in tumor initiation and progression, and in the response of HNCs to radiotherapy. In this respect, cell cycle checkpoint mechanisms constitute an important part of the DNA damage response, as they either pause the cell cycle to repair the DNA damage or, in cases where the damaged DNA cannot be repaired, they signal programmed cell death in order to minimize the transmission of DNA errors [104].

#### 4.1.1. Ataxia Telangiectasia Mutated (ATM)

ATM is a well-characterized serine-threonine kinase, primarily activated upon DSBs, which are considered the most deleterious DNA lesions invoked by replication stress [102]. A key regulator of ATM activation upon the formation of DSBs is the MRN complex (MRE11-RAD50-NBS1), which is usually responsible for the rapid localization of ATM to DSBs [102]. Although there is enough evidence to support that MRN acts as a DSB sensor, the way that it activates the ATM and ATR kinases has not been completely elucidated [102]. ATM is able to autophosphorylate, a property that promotes its association with Mdc1 and its accumulation at DSBs [102,105,106]. The activation of ATM is followed by phosphorylation of downstream molecules such as H2AX, BRCA1, Chk2, p53, and Chk2, among others, promoting DNA repair via HR, or leading the cell to apoptosis [102,107,108,109,110]. Germline inactivation of ATM leads to Ataxia–Telangiectasia Syndrome, an inherited disorder characterized by DNA repair defects and subsequent genomic instability [111]. These patients have been associated with an increased risk (one in three affected individuals) of developing HNSCC and gynaecological squamous cell carcinomas, with the onset of the disease usually occurring during early adulthood [112,113]. In addition, in certain tumors, *ATM* gene aberrations usually involve somatic mutations [114], while *ATM* promoter hypermethylation is also evident in a significant proportion of HNCs (25%), with the resulting downregulation of *ATM* expression being associated with poor prognosis [71,115,116]. Recent evidence also suggests that an impaired *ATM*-orchestrated DNA damage response, irrespectively of *ATM* expression levels, might be responsible for the radiosensitivity of HPV(+) HNSCCs, even though the exact molecular mechanism remains unknown [100]. Indeed, the interaction between ATM and the E7 viral oncoprotein has been described to induce the phosphorylation and activation of the DNA damage protein kinase CHK2 in HPV(+) cells, which is required for HPV differentiation and genome amplification [117].

#### 4.1.2. ATM- and Rad3-Related (ATR)

In contrast to ATM, ATR responds to a wide variety of DNA damage, in addition to DSBs [102,111]. ATR, along with its partner, ATRIP, are localized to sites of single-stranded DNA (ssDNA), and this may constitute the trigger that induces the ATR response in DSBs generated during the S and G2 phases of the cell cycle, i.e., resected DSBs or DSBs associated with stalled replication forks [102,111,118]. Additionally, ATR appears to be implicated in a mechanism that prevents the premature onset of mitosis, while it is activated by replication origins arising during the S phase [119]. Due to the plausible role of *ATR* in mammalian cell DNA integrity, mutation rates in this gene are quite low, with the exception of a rare genetic syndrome, the Seckel syndrome, which is characterized by biallelic *ATR* mutations [111]. Nonetheless, it has been estimated that 4.36% of patients with HNC present with *ATR* gene mutations [120].

#### 4.1.3. Chk1 and Chk2

Control of the cell cycle mainly involves two checkpoint kinases, Chk1 and Chk2, both of which are serine/threonine kinases. Chk2 is involved in all three main cell cycle checkpoints, i.e., the G1/S checkpoint, the intra-S checkpoint, and the G2/M checkpoint [121]. It is mainly activated by ATM upon DNA double-strand breakage, and activation involves autophosphorylation and dimerization [121]. Chk1 is not as stable as Chk2; its activation is restricted to the S and G2 phases [122] with the involvement of ATR in single-strand breaks (SSBs) [123]. However, ATM is also required for the activation of Chk1 in response to DSBs and for repair through homologous recombination [123].

#### 4.1.4. p53-Binding Protein 1 (53BP1)

53BP1 also participates in the activation of Chk1 and Chk2 and in the repair process [124]. Specifically, 53BP1 is rapidly recruited to DSBs and mediates their repair in mammalian cells. In addition, it is actively implicated in the protection of the replication forks during cellular replication stress responses, as defective ATR-Chk1-p53 signaling and caspase 3-mediated cell death are observed in the absence of 53BP1 [125]. During the G1 phase, 53BP1 has been reported to promote the repair of DSBs through the NHEJ mechanism and, in this way, to counteract repair via HR, i.e., through DNA end resection. Rap1-interacting factor 1 (RIF1) and Pax transactivation domain-interacting protein (PTIP) have been shown to interact with 53BP1 in an ATM-dependent manner and to act as key effectors that allow 53BP1 to block DSB resection in G1 and to promote NHEJ [126,127,128]. In contrast, during the G2-S phase, BRCA1 and CtIP have been shown to antagonize the actions of 53BP1-RIF1 and 53BP1-PTIP in order to promote HR repair; BRCA1 appears to recruit CtIP to DSBs and to promote DNA end resection, but the exact mechanism has not been elucidated yet [129,130]. Overall, it is generally accepted that 53BP1-RIF1 and 53BP1-PTIP protein complexes guide the decision toward the NHEJ repair process, whereas the BRCA1-CtIP protein complex guides the decision toward the initiation of the HR process [130].

Research on human HNC samples has also revealed a perturbed activation of DDR, or rather a defect in the synchronization of the cell cycle during DDR activation, as evidenced by elevated expression of Ki-67, a protein marker for cell proliferation, and localization of 53BP1 foci in the nucleus [131]. The researchers proposed 53BP1 expression as a potential biomarker for estimating the genomic instability invoked by DSBs or by abnormally activated DDR in oral squamous epithelial lesions [131]; this is in agreement with the results of another study highlighting the potential usefulness of 53BP1 IF analysis as a diagnostic tool for Thyroid Follicular Tumors [132]. Preclinical data have also demonstrated that 53BP1 can regulate cell cycle arrest through the modulation of *TP53*, *CHK1*, and *CHK2* gene expression in ESCC; specifically, targeted downregulation of the 53BP1 protein has been shown to cause a reduction in *p53* gene expression, but to be negatively correlated with *CHK1* and *CHK2* gene expression [133].

### 4.2. Molecules of the Homologous Recombination (HR) Mechanism

In patients with HR-related defects, such as *BRCA1* and *BRCA2* deficiency, or in patients with FA syndrome, chromosomal instability is usually observed [48,134]. When the HR process is impaired, chromosomal translocations appear when two DSBs from different chromosomes (i.e., four DNA-free ends) are aberrantly joined via NHEJ, leading to genomic rearrangements that are associated with high genomic instability and cancer [135]. Additionally, chromothripsis, which results from multiple DSBs in a single chromosome, may be followed by chromosomal segments rejoined via NHEJ, driving carcinogenesis through the loss of tumor suppressor genes or the activation of oncogenes. Thus, the error-prone nature of NHEJ and HR impairment can lead to genomic rearrangements and carcinogenesis [135]. However, there is substantial evidence to suggest that certain genetic deficiencies are associated with a higher frequency of HR, and can therefore lead to genomic instability and increase the possibility of cancer occurrence [136].

#### 4.2.1. FA Genes

Fanconi Anemia is a genetic disorder caused by the germline inactivation of one or more genes, and patients may present with bone marrow failure and a high predisposition to cancer [137]. The FA pathway is involved in ICL repair through HR-mediated mechanisms [90]. In this context, FA proteins act as DNA integrity factors through the stabilization of replication forks and the regulation of cytokinesis, in addition to their canonical role in ICL repair [90]. In FA patients, HNSCC is considered the most common type of solid cancer, associated with a 700-fold increased risk [137].

Defects in HR repair are not only implicated in the initiation of tumorigenesis, but are also associated with secondary and subsequent stages of carcinogenesis and tumor progression [136]. In sporadic HNSCC, this is evidenced by the downregulation of FA-related genes and *FANCF* gene silencing. Indeed, functional defects in DNA crosslink repair have been observed in a significant proportion of HNSCC cell lines, in terms of MMC-hypersensitivity, G2-blockade, and olaparib (PARP-inhibitor) hypersensitivity [138]. The mono-ubiquitylation of FANCD2 protein, a necessary prerequisite for the activation of the FA pathway, also appears to be compromised in certain HNC cell lines, while FA/HR-related gene variants are present in 19% of HNSCC tumors and in 24% of cell lines, and defects are associated with worse prognosis [138].

The carcinogen acetaldehyde has been shown to activate the ATR-Chk1-dependent damage response mechanisms leading to S and G2/M cell cycle arrest in esophageal epithelial cells (keratinocytes), followed by FA pathway activation-directed repair [139]. In this context, depletion of FANCD2 protein has been associated with excessive DNA damage and apoptotic cell death, suggesting that it is an important contributor to the repair of acetaldehyde-induced DNA damage in these cells [139]. Such data highlight potential mechanisms of excessive alcohol consumption-related tumorigenesis, as well as mechanisms implicated in genomic instability that increase the risk of developing SCCs in FA patients. Future studies will determine whether these mechanisms also apply to HNSCC tumors.

#### 4.2.2. BRCA1 and BRCA2

As mentioned earlier, *BRCA1* and *BRCA2* have essential functions in the HR repair pathway, and breast and ovarian tumors with defects in these genes can be successfully treated with PARP inhibitors [140]. Molecular profiling of HNSCC patient samples has identified *BRCA1* and *BRCA2* mutations with a frequency of 5.75% and 9.2%, respectively, which seem to co-exist with *TP53* and *PI3KCA* pathway mutations [141]. However, the clinical significance of most detected variants remains to be determined.

#### 4.2.3. RAD51

The RAD51 protein has essential roles in successful DSB repair via homologous recombination; it binds to ssDNA near the repair sites and exhibits DNA-dependent ATPase activity [142,143]. By catalyzing the recognition of homology and strand exchange, RAD51 contributes to re-synthesizing the lost sequence [144]. Xrcc3 (X-ray repair cross-complementing group 3; 14q32.3) is responsible for the accumulation of RAD51 at sites of DSBs in the cell nucleus and for the enzymatic resolution of the resulting Holliday junction [145].

Two *RAD51* single nucleotide polymorphisms (SNPs) have been identified as potential regulators of mRNA stability, altered translational efficiency, and the potential to induce carcinogenesis: *135GC (rs1801320)* and *172GT (rs1801321)* [146]. A case study revealed a possible association of *RAD51* (*135GC* genotype) and *XRCC3* (*722CT* and *722TT* genotypes) gene polymorphisms with an increased risk of HNSCC occurrence in the Polish population [147]. The *RAD51 135GC* polymorphism, in particular, already associated with an increased risk of developing breast cancer and acute myeloid leukemia, has also been linked to an increased risk of developing HNC in other populations, especially among smokers and drinkers [148]. Interestingly, a meta-analysis revealed that whereas the *RAD51 135GC* polymorphism is associated with a high susceptibility to HNC and esophageal cancer among Caucasians, the *G172T* polymorphism appears to confer a potential protective effect against HNC among the same population, as it is associated with a decreased risk of HNC occurrence [146,149].

In OSCC patients, RAD51 protein expression levels are significantly increased compared to healthy individuals [150]. Notably, among OSCC patients, those with lymphatic metastases appear to have significantly higher *RAD51* gene expression, suggesting that the respective protein may be involved in lymph node metastasis. Additionally, *RAD51* expression has been related to the stage of malignancy, with significantly higher levels being observed in poorly differentiated tissues, as compared to moderately or well-differentiated tissues, further highlighting its potential to be used as a biomarker for OSCC staging [150].

Increased *RAD51* mRNA expression levels are correlated with worse prognosis in HPV(+) HNSCC tumors; the combination of a RAD51 inhibitor with a Wee1 inhibitor has been shown to significantly inhibit tumor growth in mice with HPV(+) HNSCC tumors, as compared to mice with HPV(−) HNSCC, suggesting that HPV(+) tumors may be more dependent on DDR mechanisms in order to counteract the effects of increased replication stress (due to the transformation by the viral oncogenes *E6* and *E7*) [151]. In the same context, the inhibitors B02 and AZD1775 have been shown to act as radiosensitizers to HPV(−) HNSCC tumors in vitro and in vivo [151].

### 4.3. Molecules of the Non-Homologous End Joining (NHEJ) Mechanism

#### 4.3.1. DNA-Dependent Protein Kinase (DNA-PK)

DNA-PK is a serine/threonine-protein kinase that possesses a catalytic subunit (DNA-PKcs) and a Ku heterodimer, consisting of the Ku70 and Ku80 subunits; it is implicated in DDR mechanisms and other processes that are associated with the maintenance of DNA integrity, such as cell cycle progression, transcription, and telomere maintenance [152]. As with the ATM kinase, DNA-PK mainly responds to DSBs and mediates their repair. In particular, the Ku heterodimer recognizes and binds to the DSB and recruits and activates the DNA-PKcs to form the active DNA-PK complex, in this way promoting the NHEJ DNA repair mechanism [135]. Recent evidence suggests that DNA-PK is also involved in a complex regulatory network for the pathway choice between HR and NHEJ [152,153], while phosphorylation of T2609 and T3950 residues is implicated in radioresistance [153]. Interestingly, while mutational inactivation of DNA-PKcs has been shown to impair NHEJ, subsequently leading to DNA repair through HR, drug inhibition of DNA-PKcs has been shown to impair both pathways [152,154]. Overall, DNA-PKcs is implicated in cancer progression and metastasis [155]. In the context of HNCs, DNA-PK overexpression appears to be significantly associated with decreased survival in NPC patients, suggesting that it may be used as a prognostic biomarker for this particular HNC subtype [156]. In addition, DNA-PKcs overexpression has been associated with poor outcomes in patients with NPC undergoing intensity-modulated radiotherapy, suggesting an additional role in predicting response to therapy [157]. Notably, DNA-PK expression has been shown to increase following irradiation of OSCC, with the rate of increase being directly correlated with therapeutic resistance [158], and for this reason, several DNA-PK inhibitors are currently being investigated for their radiosensitizing properties [159].

#### 4.3.2. Ku Protein

When HR mechanisms are not available, as for example outside of the S and G phases of the cell cycle or due to defects in key repair proteins, DSBs are predominantly repaired via the NHEJ mechanism. The Ku protein constitutes an essential player in this process, being a nuclear DNA-binding protein that recognizes DSBs and facilitates their repair [160]. Ku consists of two subunits, one that is approximately 70 kDa and another that is 80 kDa (Ku70 and Ku80, respectively) [161]. Overexpression of Ku80 is associated with an adverse prognosis in ESCC [162], a cancer type that is not classified as HNC but which is often accompanied by head and neck second primary tumors (in approximately 7% of ESCCs patients) [163]. In vitro experiments on both radiosensitive and radioresistant HNC cell lines have revealed that *ku70/80* gene overexpression is associated with radioresistance and may therefore have some utility as a potential predictive marker of radioresistance in HNCs [160]. Additionally, siRNA-silencing of β-catenin in the radiosensitive AMC-HN-9 cell line has been shown to impair the radiation-induced overexpression of Ku80 in an AMPK signaling pathway-dependent manner [164]. *Ku80* mRNA expression has also been negatively associated with the expression of checkpoint molecule PD-L1 (programmed cell death-1 ligand) in many cancer types, including HNCs, following irradiation [165]. Such observations provide substantial evidence that the NHEJ mechanism is implicated in the repair of DNA damage invoked by radiation and emphasize the necessity of discovering new NHEJ targets for chemotherapeutic radiosensitization of head and neck tumors.

#### 4.3.3. PARP Molecules

PARP1 (Poly (ADP-ribose) polymerase-1) is another sensor known to detect DSBs; however, despite being involved in many cellular processes, such as cell proliferation, differentiation, the repair process of the SSBs and DSBs through different repair pathways, the stabilization of DNA replication forks and in the modification of chromatin structure, its implication in NHEJ is not fully understood [161]. Nonetheless, it has been observed that PARP1 and the Ku70/80 complex localize to DNA lesions considerably earlier than other DSB sensors, that PARP1 competes with the Ku70/80 complex at the DSBs in the S/G2 phases, and that the Ku complex can be replaced by PARP1 at DSB sites [166]. On the contrary, the Ku70/80 complex appears to occupy the DSB sites exclusively in the G1 phase, thereby suggesting that each sensor recognizes DSBs at different stages of the cell cycle; in addition, it has been postulated that PARP 1 may act as a regulator of the Ku70/80 complex in DSBs in the S/G2 phases, as it has been shown to enzymatically remove the Ku complex from these sites [166].

In addition, PARP5A and PARP5B are PARP molecules that also catalyze poly (ADP- ribose) PAR chains, but their role in cancer is still not fully deciphered. While PARP5B downregulation has been observed in prostate, breast, and HNCs [167,168], recent data demonstrate that null PARP5B tumor cells are characterized by an impairment of the NHEJ mechanism, and that treatment with a PARP5B inhibitor combined with low doses of etoposide drives SCC human cell lines into senescence and apoptosis [169]. The latter seems to be dependent on the actions of the NBS1 protein, which constitutes an essential component of the MRN (MRE11-RAD50-NBS1) complex; by accumulating the MRN complex at damaged sites, NBS1 amplifies ATM activation, which in turn activates and orchestrates DSB repair [170]. In another study, the consequential reduction in NSB1 expression following PARP5B inhibition was associated with the activation of ATR signaling, which in turn led to senescence and apoptosis, highlighting PARP5B as a potential target for chemotherapeutic intervention in HNSCC [169].

Preclinical data from HNSCC cell lines and mouse models have demonstrated that *NSB1* gene downregulation is associated with disruption of HR and increased sensitivity to PARP inhibitors; in addition, co-inhibition of the MRN complex and PARP has been shown to induce DSB accumulation, telomere shortening, and ultimately cell death [167]. Such observations indicate that simultaneous inhibition of genes/molecules that participate in two or more DNA repair pathways may induce a cytotoxic response that has been described as synthetic lethality [167,171]. The latter only happens in the presence of another specific defect, in this case, a defect in a DNA repair gene, and is best exemplified with the use of PARP inhibitors in cancer cells carrying defects in *BRCA1* or *BRCA2* genes [172]. In the context of *BRCA1/BRCA2* deficient cells, PARP inhibition tends to accumulate SSBs arising during normal cellular activity and to result in DSBs that are formed through the collision of SSBs with a DNA replication fork; these DSBs require HR repair that cannot take place in the absence of fully functional BRCA1/BRCA2 proteins and ultimately induce cell death [172,173]. Interestingly, the observation that PARP inhibition in HR deficient cells can still lead to synthetic lethality but may not be necessarily accompanied by SSB accumulation has led to the conclusion that PARP1 must also be actively implicated in the Alt-NHEJ repair process, which is believed to take place in HR- and/or NHEJ-defective cancer cells [94]. This could have tremendous implications for the development of therapeutic synthetic lethality approaches in malignancies carrying defects in the two major DSB repair pathways, including HNCs [174,175].

### 4.4. Molecules of the Nucleotide-Excision Repair (NER), Base-Excision Repair (BER), and Mismatch Repair (MMR) Mechanisms

#### 4.4.1. NER-Associated Genes

These are genes that appear to be downregulated in HNSCC patients and include *ERCC1*, *ERCC2/XPD*, *XPA*, and *XPC* [11]. Low expression levels of *ERCC3* and *XPA* in lymphoblastoid cells and low levels of *XPB* in lymphocytes are commonly observed in HNSCC patients and have been associated with an increased risk of developing the disease [176]. In addition, NER-associated genes have been proposed as biomarkers with potential value in predicting treatment efficacy and outcome in HNSCC [177]. However, while certain studies have found an association between *ERCC1* and *XPA* upregulation with poor OS in patients with OSCC, others have noted better OS [178]. Notably, these genes have been proposed as a biomarker for the prediction of 5-FU and cisplatin-based chemotherapy in HNSCC patients [179,180].

#### 4.4.2. BER-Associated Genes

*APEX1* and *XRCC-1* have recently been found to be downregulated in patients with HNSCC [11]. Overexpression of these genes has been correlated with better clinical staging and negative lymph node metastasis in patients with OTSCC [179,181].

#### 4.4.3. MMR-Associated Genes

Downregulation of *MLH1*, *MSH2*, *MLH3*, and *PMS2* genes, all key molecules of the MMR mechanism, has been associated with reduced DNA repair capacity, development of malignant lesions in OSCC, and tobacco-induced methylation in smokers [179].

Interestingly, a prognostic 13-DNA-repair-gene signature has recently been identified and shown to accurately and independently predict the clinical outcome of patients with HNSCC [182]; the signature includes the *MORF4L2*, *COPS2*, *USP10*, *WAS*, *UVSSA*, *PRRX1*, *ZBTB1*, *DCLRE1C, MSH5, DOT1L*, *ZBTB7A*, *POLR2C,* and *MORF4L1* genes, known to be involved in a variety of DNA repair pathways, including NER, BER, MMR, and DSB repair [182,183].

**Figure 1:**
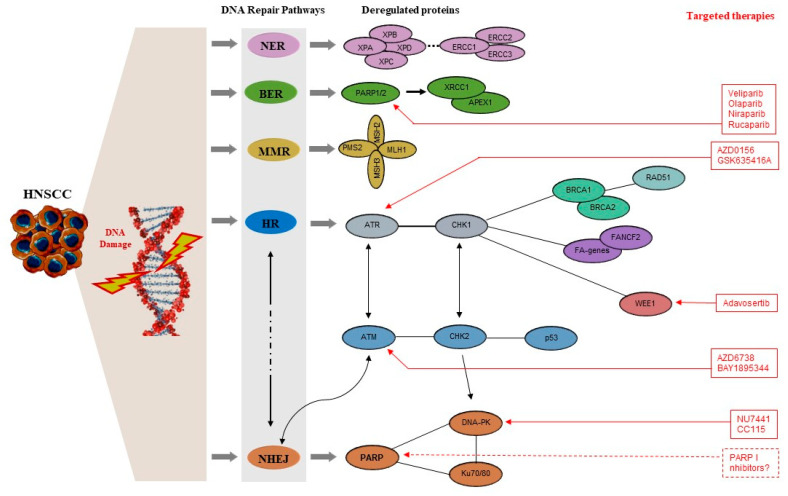
Schematic representation of the main DNA repair pathways, the proteins involved in each pathway that are deregulated in HNCs, and the inhibitors that are being investigated as targeted therapeutics. The clarification of the complex interactions between DDR genes with altered expression in HNC can reveal potential biomarkers for predicting clinical outcome and for guiding therapy selection. The investigation of the complex interplay between the different DNA repair mechanisms, and how these are affected by the altered expression of key proteins in HNC, can significantly contribute to the development of new inhibitors for targeted therapy. APEX1: Apurinic/Apyrimidinic Endodeoxyribonuclease 1; ATM: Ataxia Telangiectasia Mutated; ATR: Ataxia Telangiectasia and Rad3-related; BER: Base Excision Repair; BRCA1: Breast Cancer gene 1; BRCA2: Breast Cancer gene 2; CDKi: Cyclin-Dependent Kinase inhibitor; CHK1: Checkpoint kinase 1; CHK2: Checkpoint kinase 2; DDR: DNA damage response; DNA-PKi: DNA-dependent Protein Kinase inhibitor; DSBs: double-strand breaks; ERCC1: Excision Repair Cross-Complementing Rodent Repair Deficiency, Complementation Group 1; ERCC2: excision repair cross-complementing rodent repair deficiency Gene 2; ERCC3: excision repair cross-complementing rodent repair deficiency Gene 3; FA-genes: Fanconi Anemia genes; FANCD2: Fanconi Anemia Complementation Group D2; HNC: head and neck cancer; HNSCC: Head and Neck Squamous Cell Carcinoma; HR: Homologous Recombination; MLH1: MutL protein homolog 1; MMR: Mismatch Repair; MSH2: MutS homolog 2; MSH3: MutS Homolog 3; NER: Nucleotide Excision Repair; NHEJ: Nonhomologous End Joining; PARP1/2: Poly(ADP-Ribose) Polymerase 1/2; 2PARPi: Poly (ADP-ribose) Polymerase inhibitor; PMS2: Postmeiotic Segregation Increased 2; SSBs: single-strand breaks; XPA: Xeroderma pigmentosum complementation group A; XPB: xeroderma pigmentosum, complementation group B; XPC: Xeroderma pigmentosum, complementation group C; XPD: Xeroderma Pigmentosum, complementation Group D; XRCC1: X-ray Repair Cross Complementing protein 1.

Figure 1 schematically represents the DNA repair pathways that are implicated in the pathogenesis of HNC and the proteins that have been found to be deregulated in these pathways.

### 4.5. Genes That Have Been Functionally Related to HPV(+) HNSCCs

HPV oncogenes *E6* and *E7* are responsible for tumor initiation and immortalization, and are known to deregulate the onco-suppressor gene *TP53* and retinoblastoma protein (pRb), thereby leading to deregulation of the cell cycle and inhibition of apoptosis [184,185,186]. Relevant information from the TCGA database indicates that a great proportion of oropharyngeal and tonsil tumors are HPV(+) without *TP53* gene mutations, while HNSCCs in other sites are more prone to developing *TP53* mutations and appear to have lower rates of HPV infection [82]. This inverse relationship may reflect the important role of the viral oncoproteins E6 and E7 in oropharyngeal and tonsil HPV-related tumorigenesis [82]. On the other hand, *TP53* gene mutations are implicated in HPV(−) HNC tumorigenesis, as evidenced through experiments in HNC cell lines and animal models; *E7* has been shown to increase the radiation-induced DNA damage, to decrease the sub-lethal DNA damage repair, and to lead to overexpression of the HR-related protein RAD51 [187].

In addition, current evidence suggests that HPV-associated tumors exhibit higher radiosensitivity as compared to HPV(−) cell lines through a DDR-dependent manner. Specifically, Jonathan E. Leeman et al. demonstrated that the HPV16 E7 oncoprotein leads to the suppression of cNHEJ and promotes the MMEJ mechanism, which is considered to be error-prone and highly mutagenic [78]. In addition, HPV(+) HNSCCs appear to be deficient in TGF-β signaling, which is associated with a perturbed HR function and increased reliance on Alt-NHEJ for repair [188].

The presence of HPV infection in HNCs has also been correlated with overexpression of the *p16* gene, and this may result from pRb inactivation via the E7 HPV protein [189]. P16 protein constitutes an important cell cycle regulator, acting as a CDK inhibitor, and for this reason, alterations in *p16* gene expression are frequently observed in several malignancies [189]. Nonetheless, HPV(−) malignancies characterized by *p16* overexpression have also been identified [190]. Currently, the evaluation of *p16* expression is used as a surrogate marker for HPV infection in HNCs, but the data regarding its diagnostic value are rather contradictive. While a great proportion of oropharyngeal squamous cell carcinomas (OPSCCs), especially tonsillar and tongue base carcinomas (TSCC/BOTSCC), appear to contain the HPV genome and evaluation of *p16* gene expression has successfully been used for the estimation of prognosis and recurrence of the disease [191], some researchers propose the cautious use of *p16* expression status for the diagnosis of HPV(+) HNSCCs in non-tonsillar, non-base of tongue oropharyngeal cancers [192]. In OSCCs such as oral tongue cancers, for example, HPV infection and *p16* overexpression do not seem to coincide [193]. Other studies indicate that HPV infection in OSCCs does not affect the survival outcome of the disease [194] and that *p16* overexpression may be the result of other pathways or cellular mechanisms and not the consequence of HPV infection [193]. However, additional large-scale studies need to be performed in order to draw conclusions on the diagnostic role of *p16* expression in the different types of HNCs.

Table 1 summarizes the genes involved in the DDR pathways and how these are deregulated in the pathogenesis of HNC.

## 5. Novel Therapeutic Targets: Preclinical and Clinical Evidence

The significant inter- and intratumoral heterogeneity of HNSCCs make targeted therapy a real challenge. Current treatment approaches include surgery, radiation therapy, chemotherapy, targeted therapy, and immunotherapy or combinations of these treatment modalities [196]. One of the most common treatment options is photon-based radiotherapy (XRT), either alone or with chemotherapy agents such as methotrexate, platinum compounds with or without 5-fluorouracil, and cisplatin [196,197,198]. However, treatment approaches often have severe side effects with unfavorable patient outcomes (i.e., the 5-year overall survival rate after XRT treatment does not exceed 40% for HPV(−) OSCC stages III to IV) [199]. Successful treatment is usually impeded by tumor-related mechanisms which repair the radiotherapy- and/or chemotherapy-induced DNA damage, leading to radioresistance and/or chemoresistance of the tumor [196]. Consequently, there is a need for new effective treatment strategies with the lowest possible toxicity. When it comes to DNA damage response mechanisms, efforts for successful therapy have concentrated on the design of new targeted drugs and on the identification of appropriate drug and radiotherapy combinations, based on specific molecular characteristics of the tumor, in order to generate lethal DNA damage, such as DSBs, and prevent their repair, leading to cell death. Table 2 includes a list of DDR inhibitors that are currently being tested in clinical trials, whereas Table 3 includes the DDR inhibitors that are being investigated at the pre-clinical stage.

### 5.1. PARP Inhibitors

The most attractive therapeutic targets are poly (ADP-ribose) polymerases (PARPs), as these are implicated in the regulation of more than one DNA repair pathway (BER, NER, Alt-NHEJ, NHEJ, MMEJ) [200]. As mentioned earlier, PARP inhibition was first described in cancer cells with defective BRCA1/BRCA2 protein function, and then clinical trials confirmed the effectiveness of PARP inhibitors as monotherapy in patients with *BRCA1(−*/*−)* and *BRCA2(−*/*−)* tumors [196]. PARP inhibition was the first targeted therapy of synthetic lethality, based on the observation that inhibition of the BER mechanism leads to the formation and accumulation of SSBs and to the collapse of replication forks in highly replicating cancer cells, with subsequent development of DSBs that cannot be repaired through the high-fidelity HR mechanism in homozygous BRCA-defective cells, leading to apoptosis [195,196,201]. However, more recent studies indicate a potential role of PARPs in NHEJ, and propose that it is the deregulation of NHEJ that leads to synthetic lethality in BRCA2-deficient tumors, as experimental inhibition of key BER proteins in these tumors did not appear to have the same effect on PARP inhibition [202,203,204,205]. In addition to tumors with *BRCA* mutations, cells bearing HR protein deficiencies, for example ATM deficiency, also appear sensitive to PARP inhibition [196]. This observation supports the notion that PARP inhibitors may be effective in tumors bearing a variety of deficiencies in HR and other DDR mechanisms, which are estimated to constitute approximately 40% of HNC tumors [196].

Interestingly, PARP inhibitors are currently being tested in combination with chemotherapeutic agents, as well as with other inhibitors (i.e., EGFR inhibitors, as a great percentage of HNCs accumulate EGFR mutations) and with radiotherapy. Preclinical testing of the PARP inhibitor veliparib (ABT-888) in HNC cell lines and an HPV(+) patient xenograft has produced encouraging results; HPV(+) HNSCC cell lines that were deficient for BRCA2 and DNA-PK proteins, both key molecules of HR and NHEJ, exhibited sensitivity to the PARP inhibitor, as evidenced by a decreased survival rate in vitro and a delay in tumor growth in vivo [206]. Recently, the results of a phase I trial evaluating the maximum tolerated dose (MTD) and safety of veliparib in combination with carboplatin and paclitaxel induction chemotherapy (IC) for locoregionally advanced HNSCC demonstrated that veliparib is well tolerated, with promising survival data following an adequate follow-up interval [207].

On the other hand, HNSCC cell lines with *SMAD4* homozygous deletion have been shown to exhibit sensitivity to olaparib, a well-studied PARP inhibitor, which appears to increase DNA damage and cell death in combination with RT in vivo [208]. In a phase I clinical trial, five out of six *SMAD4*-negative HNSCCs and four out of eight *SMAD4*-positive HNSCCs responded to standard treatment plus olaparib [208]. A recent study has highlighted the importance of PARP-1 in regulating the repair of complex DNA damage (CDD) induced by high-LET radiation by demonstrating that high-LET proton-induced CDD and concomitant inhibition of PARP-1 (with olaparib) leads to cell death in the HPV(−) HNSCC cell line UMSCC74A [209].

Another study on two HPV(+) and two HPV(−) HNSCC cell lines revealed that the PARP inhibitor niraparib enhanced their sensitivity to both photon and proton radiotherapy and increased the relative biological effectiveness (RBE) of protons, possibly by inhibiting DDR [210]. In addition, the combination of niraparib with the Chk1 inhibitor MK-8776 has been shown to enhance the radiosensitivity of HPV(+) HNSCC cells, whereas co-administration with the Wee1 inhibitor MK-1775 seems to further enhance the radiosensitivity of HPV(−) HNSCC cells in vitro [211]. An ongoing phase II study is currently evaluating the combination of niraparib with PD-1 blockade (dostarlimab) in patients with recurrent/metastatic HNSCC, with primary endpoints including stable disease, partial or complete response, and secondary endpoints including adverse events, progression-free survival (PFS) and overall survival (OS) [212].

### 5.2. Immune Checkpoint Inhibitors

PD-1 and its associated ligand, PD-L1 (Programmed death ligand 1), both constitute immune checkpoint molecules expressed on the surface of T-cells and cancer cells, respectively. The binding of PD-1 to PD-L1 produces a negative signal that leads to the inactivation of T cells and allows immune evasion of tumor cells, leading to treatment failure; therefore, inhibition of these molecules with specific antibodies (ICIs), represents an attractive therapeutic strategy [213]. A recent meta-analysis revealed that the use of anti-PD-1 monoclonal antibodies is more effective in smokers and in HPV(−) patients, whilst anti-PD-L1-based therapy appears to be more efficient in female patients with locally recurrent HNC and in HPV(+) patients [214]. Despite the fact that this type of immunotherapy appears to offer a therapeutic advantage in terms of tumor progression control and OS compared to chemotherapy, only a small subset of HNSCC patients really benefit from ICI treatment [214]. Current data demonstrate that despite the PD-L1 protein being over-expressed in over 50% of HNSCC tissues, only 15% of patients will respond to PD-1/PD-L1 inhibitors [214]. Nonetheless, such strategies may provide an alternative solution in cases of advanced disease where traditional therapy is not effective or where the therapeutic effect may be enhanced through a combination with other treatment approaches.

As mentioned earlier, EGFR is another molecular target for which monoclonal antibodies have been developed. The EGFR is a cell surface protein that binds to the Epidermal Growth Factor, activating downstream signaling cascades leading to DNA replication, cell proliferation, and differentiation [215]. Abnormal *EGFR* gene expression is associated with tumor progression in HNC, with overexpression being observed in approximately 90% of HNSCCs [196,216]. EGFR protein signaling is actively implicated in DDR mechanisms, while deregulation of this process has been linked to resistance to chemo and/or radiotherapy [217]. As a result, monoclonal antibodies against EGFR aim to downregulate its overexpression; cetuximab is currently the only FDA-approved anti-EGFR agent used for HNC treatment, whereas panitumumab, another EGFR-specific antibody, is under clinical evaluation for its effectiveness in patients with advanced HNSCC [218]. The response rate of HNSCCs to cetuximab has been estimated at approximately 20%, regardless of HPV status and EGFR overexpression [219]. Current investigations are focused on combination treatments that include EGFR and PARP inhibitors. The combination of cetuximab and olaparib with radiation seems to induce an increase in DNA lesions, senescence, apoptosis, and control of tumor growth both in vitro and in vivo [220]. The proposed mechanism behind these observations is that the combination inhibits the NHEJ-mediated DNA repair and induces senescence through p21 signaling in BRCA1/2 proficient HNSCC cells. The safety of this triple combination has previously been evaluated in a phase I clinical trial [221].

### 5.3. Tyrosine Kinase Inhibitors

Targeted therapy against EGFR can also be accomplished with Tyrosine Kinase Inhibitors (TKIs), which antagonize ATP binding to the tyrosine–kinase intracellular domain of EGFR [222]. In HNC clinical trials, reversible EGFR TKIs, such as gefitinib and erlotinib, do not seem to confer a therapeutic benefit either as monotherapy or in combination with radiation treatment, with the response rate of erlotinib estimated at 10–15% in HNSCC patients [223]; on the other hand, multitarget TKIs such as lapatinib (reversible dual EGFR and HER2 TKI), afatinib, and dacomitinib (both irreversible EGFR, HER2, and HER4 pan-HER TKIs) have shown promising results in clinical trials, in terms of improving the objective response rate in patients with HNSCC [216,224,225]. As HNSCC is a highly heterogeneous disease and targeted EGFR monotherapy has produced moderate results in treatment efficacy, and in some cases has even led to therapeutic resistance [196,216], future studies are expected to concentrate on the development of combination therapies. Despite the heterogeneous mutational landscape of HNSCCs, the *TP53* gene is mutated in approximately 72% of tumors according to the TCGA database, thereby highlighting the respective p53 protein as a potential biomarker and a promising target for more effective treatment. In this context, it has already been reported that NHSCCs with *TP53* mutations or HPV(+) tumors with aberrant *TP53* expression are sensitive to a Wee1 inhibitor, AZD1775, either used as a single agent or in combination with other chemotherapeutic agents [226]. A recent phase I clinical trial investigating AZD1775 in combination with CDDP (cisplatin) and docetaxel in HNSCC patients has so far produced encouraging anti-tumor efficacy in patients with defects in *TP53* gene expression [227].

Wee1 normally acts as an inhibitor of CDK1 and CDK2, playing a crucial role in the G2/M checkpoint of the cell cycle. In addition, Wee1 may be implicated in the cell-size checkpoint, in the sense that it prevents entry into mitosis before the cells have reached a specific size [228]. Upon excessive DNA damage, this may lead to a prolonged G2 phase, as the G2 checkpoint is responsible for repairing DNA damage [229]. Consequently, inhibition of Wee1, the principal gatekeeper of this checkpoint, represents a promising strategy for the treatment of HNSCC, as it may lead to overactivation of CDK1/2, increased replication stress, abrogation of DNA damage checkpoints and cleavage of stalled replication forks by the abnormally activated MUS81 and SLX4 endonuclease complex [228,230]. Recent data support the notion that the effectiveness of Wee1 inhibitors relies on an interplay between replication stress and cell cycle checkpoint failure, as evidenced through combined inhibition of PARP and Wee1, which appears to confer tumor-specific radiosensitization of HPV(+) HNSCC cells [174].

### 5.4. CDK Inhibitors

CDK inhibitors, by preventing or counteracting tumor resistance mechanisms to chemo- or radiotherapy, may also provide an efficacious alternative targeted therapy to HNC patients. Even though pan-CDK inhibitors have not been approved for clinical use due to severe side effects and low specificity, selective CDK inhibitors are currently being used in clinical practice or are under clinical evaluation [189,231]. Ribociclib, a CDK4/6i, has been shown to be effective in an HNSCC HPV(−) cell line, whereas cancer cells with endothelial mesenchymal transition (EMT) features and low expression of Rb appear to be less sensitive to it [232]. Additionally, both ribociclib and palbociclib (another CDK4/6 inhibitor) have been shown to enhance radiosensitivity in HNSCCs [189], and are therefore currently being investigated in combination with other inhibitors in recurrent, metastatic, and chemotherapy-resistant HNCs [233,234]. Further optimization is required for both pan- and selective CDK inhibitors in order to diminish unwanted side effects, and in this process it is essential to identify predictive biomarkers of effective CDK inhibition as well as optimum treatment combinations.

### 5.5. DNA-PK Inhibitors

DNA-PK inhibitors constitute another promising target for achieving more effective cancer treatment. As mentioned earlier in this review, in addition to actively contributing to the NHEJ process, DNA-PKcs is implicated in many cellular processes, such as innate immunity, pro-inflammatory signaling, metabolism, and other tumor-associated processes. To date, pre-clinical evaluation of DNA-PK inhibitors has produced more promising results in the radiosensitization of HNSCC lines, as compared to the clinically available PARP1 inhibitors, while the combination of PARP1 and DNA-PK inhibitors has been shown to enhance HPV(−) HNSCC inhibition in both mouse xenografts and cell culture, highlighting a potential clinical application in patients with HPV(−) HNSCC [235]. Indeed, combined administration of a DNA-PK inhibitor (NU7441) with olaparib and radiotherapy has been shown to inhibit tumor cell proliferation both in vivo and in vitro, further suggesting that the combination of these agents may be responsible for the inhibition of the CDK and ERK pathway-related kinases [235]. In addition, combined administration of the dual mTOR/DNA-PK Inhibitor CC-115 and radiotherapy has been shown to inhibit tumor growth and significantly reduce the migration rate in HNSCC cell lines [236].

### 5.6. ATM and ATR Inhibitors

Based on the role of ATM and ATR in DDR responses, inhibitors of these molecules are currently being investigated in several preclinical and clinical studies [175,237]. The ATR inhibitor AZD6738 appears to promote cisplatin sensitization and to increase apoptosis signaling, DNA damage, and radiosensitization that is correlated with CHK1-mediated abrogation of G2/M-arrest in HPV(+) and HPV(−) HNSCCs, as shown in both in vivo and in vitro studies [238,239]. The ATR inhibitor BAY 1895344 has also been shown to confer radiosensitization in preclinical studies of HNSCC, by enhancing radiotherapy-induced inflammation in the tumor microenvironment [240]. This process may be driven by the actions of NK cells and can be further enhanced through combination treatment with TIGIT and PD-1 targeted immunotherapy [241]. Such data emphasize the importance of clinical trials in validating the safety and efficacy of BAY 1895344 in clinical practice [242]. On the other hand, inhibition of ATM has been shown to reduce cell migration [243], while concomitant administration of AZD0156 (ATM inhibitor) and radiotherapy appears to reduce tumor growth and migration rate in both HPV(+) and HPV(−) HNSCC cell lines [236]. Additionally, the novel ATM inhibitor GSK635416A, in combination with olaparib, seems to effectively radiosensitize HNSCC cell lines, whereas GSK635416A as monotherapy effectively inhibits HR and is less cytotoxic to normal fibroblasts as compared to olaparib [244]. Such observations highlight GSK635416A as a potential radiosensitizing drug, and additional studies are warranted in order to confirm its clinical significance.

**Figure 2:**
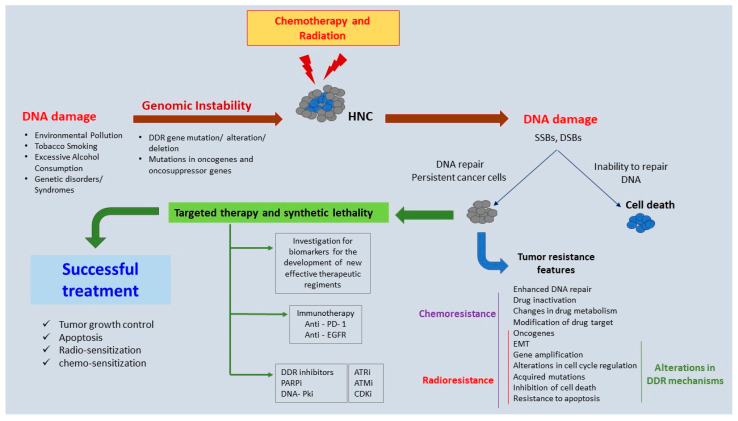
Overview of the DDR process in HNC pathogenesis, therapeutic resistance, and successful treatment. Genomic instability in HNC is believed to result from the interplay between genetic and environmental factors, and is evidenced by an ever-increasing accumulation of DNA damage, impairment of the DDR mechanism, and a highly unstable genetic environment that is driven by mutagenic stress. On the other hand, tumors that are able to effectively repair the DNA lesions (SSBs and DSBs) induced by chemotherapy and radiation are resistant to these types of treatment and exhibit altered DDR profiles. The identification of these altered DDR-related genes and pathways holds the key to overcoming resistance mechanisms and improving therapeutic outcomes in HNC. Anti-EGFR: Epidermal Growth Factor Receptor antibody; Anti-PD-1: Programmed Death-1 antibody; ATRi: ataxia telangiectasia and Rad3-related inhibitor; ATMi: ataxia telangiectasia mutated inhibitor; CDKi: Cyclin-Dependent Kinase inhibitor; DDR: DNA damage response; DNA-PKi: DNA-dependent Protein Kinase inhibitor; DSBs: double-strand breaks; EMT: endothelial mesenchymal transition; HNC: head and neck cancer; PARPi: Poly (ADP-ribose) Polymerase inhibitor; SSBs: single-strand breaks.

Figure 2 summarizes the DDR process in HNC and how this may result in therapeutic resistance or successful therapy, through the targeting of specific DDR molecules.

## 6. Conclusions

The complexity of HNC mandates a multidirectional approach for effective treatment. Pre-clinical and clinical studies are required to improve our understanding of the mechanisms implicated in HNC initiation and progression. In this regard, the investigation of the mechanisms underlying the development of chemo- and radio-resistance is expected to shed new light on alternative therapeutic combinations that will help to overcome the obstacles that arise from traditional anti-cancer modalities.

Among the best-known tumor-related mechanisms of resistance to therapy are enhanced DNA repair of the lesions invoked by chemo- and radiotherapy, gene amplification, inhibition of cell death, and resistance to apoptosis, as well as by alterations in cell cycle regulation and immune evasion. These obstacles can be overcome through the introduction of DDR inhibitors and of synthetic lethality treatments that will prevent the activation of alternative pathways. Similarly, the mechanisms employed by tumors to achieve immune evasion can be counteracted with the use of ICIs.

The increasing amount of data regarding the DDR pathways that are implicated in cancer development and progression, along with the investigation of gene polymorphisms associated with tumor initiation and progression, and the identification of specific gene mutations that correlate with therapeutic resistance, aim toward the establishment of reliable biomarkers for the timely prediction of tumor response to therapy. However, the development of new and effective treatment combinations demands not only the acquisition of new information through continuous research, but also the appropriate implementation of this information with existing knowledge in the field. Inhibitors that do not appear to be therapeutically beneficial as monotherapy could still offer a therapeutic advantage in combination treatments for a number of patients with specific tumor characteristics.

With the mechanisms of tumorigenesis, tumor resistance, and metastasis continuously being unraveled, research efforts are gradually shifting toward biomarker-based patient selection for anticancer therapy. Improved understanding and clarification of the DNA repair efficiency in HNC hold enormous potential for efficient identification and characterization of the genomic instability biomarkers that will guide therapeutic development and improve responses in cases with metastatic or resistant disease.

## Figures and Tables

**Table 1:** List of genes that are deregulated in the DDR pathways in HNC and related clinical effects.

Gene	Pathway	Abnormal Expression/Alteration	Clinical Effect	Reference
*Ku70/80*	NHEJ	Overexpression	Radioresistance	[160]
*PARP5B*	NHEJ	Downregulation/null	Impairment of NHEJ	[169]
*DNA-PKcs*	NHEJ	Overexpression	Decreased survival in NPC patients/poor outcomes in NPC patients undergoing intensity-modulated radiotherapy—potential biomarker for prediction of the response to therapy	[156,157]
*RAD51*	HR	Overexpression	Lymph node metastasis, poorly differentiated tissues, worse prognosis in OSCC	[150]
*BRCA2*	HR	Deficiency	Sensitivity to the PARP inhibitors	[195]
*BRCA1*	HR	Downregulation	Poor outcome	[71]
*FANCF* and FA-related genes	Fanconi anemia/HR	Silencing or downregulation	MMC-hypersensitivity, G2-blockade, and olaparib (PARP-inhibitor) hypersensitivity	[138]
*ATM*	DNA repair (HR, NHEJ), cell cycle arrest, apoptosis	Downregulation	Poor prognosis	[71]
*ATR*	DNA repair (HR, NHEJ), cell cycle progression, apoptosis	Mutation	Unknown	[120]
*TP53*	p53 pathway HR NHEJ	Deficiency	Short survival time and tumor resistance to radiotherapy and chemotherapy in HNSCC patients	[82]
*XPB*	NER	Downregulation	Increased risk for HNSCCs	[176]
*ERCC1*	NER	Upregulation	Enhanced response to chemotherapy	[179]
*XPA* and *ERCC1*	NER	Upregulation	Poor OS in patients with OSCC/exhibited better OS in patients with oropharyngeal SCC	[178]
*XRCC1*	BER	Upregulation	Better clinical staging of OTSCC and negative lymph node metastasis	[181]
*MLH1*, *MSH2*, *MLH3* and *PMS2*	MMR	Downregulation	Reduced DNA repair capacity in OSCCs	[179]

ATM: Ataxia Telangiectasia Mutated; ATR: Ataxia Telangiectasia and Rad3-related; BER: Base Excision Repair; BRCA1: Breast Cancer gene 1; BRCA2: Breast Cancer gene 2; DDR: DNA damage response; DNA-PK: DNA-dependent Protein Kinase; ERCC1: Excision Repair Cross-Complementing Rodent Repair Deficiency, Complementation Group 1; FA-genes: Fanconi Anemia genes; FANCF: Fanconi Anemia Complementation Group F; HNC: head and neck cancer; HR: Homologous Recombination; MLH1: MutL protein homolog 1; MMR: Mismatch Repair; MSH2: MutS homolog 2; MSH3: MutS Homolog 3; NER: Nucleotide Excision Repair; NHEJ: Nonhomologous End Joining; PARP1/2: Poly(ADP-Ribose) Polymerase 1/2; PMS2: Postmeiotic Segregation Increased 2; XPA: Xeroderma pigmentosum complementation group A; XRCC1: X-ray Repair Cross Complementing protein 1.

**Table 2:** List of DDR inhibitors currently being tested in clinical trials.

Inhibitors	Target	Combination with Chemo or Radiotherapy	Cancer Type	Clinical Trial	Clinical Trial Stage
Veliparib (ABT-888)	PARP	Carboplatin + Paclitaxel	Locoregionally advanced HNSCCs	NCT01366144	Phase I
Olaparib	PARP	Radiation	SMAD4-negative HNSCC	NCT02229656	Phase I
Niraparib + Dostarlimab	PARP + PD-1		Recurrent/metastatic HNSCC	NCT04681469	Phase I (recruiting)
Niraparib + Dostarlimab	PARP + PD-1		Recurrent/metastatic HNSCC	NCT04313504	Phase II
Olaparib + Cetuximab	PARP + EGFR	Radiation	Heavy smokers with locally advanced HNC	NCT01758731	Phase I
Panitumumab	EGFR	Platinum	Advanced HNSCC	NCT02643056	Phase II
Afatinib + Pembrolizumab	EGFR + PD-1		Recurrent or metastatic HNSCC	NCT03695510	Phase II
AZD1775	Wee1	Cisplatin + Docetaxel	Locally advanced HNSCC	NCT02508246	Phase I
MSC2490484A	DNA-PK	Radiation + Cisplatin	Locally advanced solid tumors	NCT02516813	Phase I
CC-115	Dual mTOR/DNA-PK inhibitor		Advanced solid tumors	NCT01353625	Phase Ia/Ib
BAY 1895344 + Pembrolizumab	ATR + PD-1	Radiation	Recurrent HNC	NCT04576091	Phase I

ATR: ataxia telangiectasia and Rad3-related; DNA-PK: DNA-dependent Protein Kinase; EGFR: Epidermal Growth Factor Receptor; HNC: head and neck cancer; HNSCC: head and neck squamous cell carcinoma; mTOR: mammalian target of rapamycin; PARP: Poly (ADP-ribose) Polymerase; PD-1: Programmed Death-1, SMAD4: SMA- and MAD-related protein 4.

**Table 3:** List of DDR inhibitors currently being investigated pre-clinically.

Inhibitor	Target	Concomittant Administration of Radio and/or Chemotherapy	Cancer Type
NU7441 + Olaparib	DNA-PK + PARP	Radiotherapy	HPV(−) HNSCC cell lines/mouse models
AZD1775 (Adavosertib)	Wee1	-	TP53-mutated or HPV(+) tumors
CC-115	Dual mTOR/ DNA-PK inhibitor	Radiotherapy	HPV(+) and HPV(−) HNSCC cell lines
AZD6738	ATR	Radiotherapy + Cisplatin	HPV(+) and HPV(−) HNSCC cell lines
AZD0156	ATM	Radiotherapy	HPV(+) and HPV(−) HNSCC cell lines
GSK635416A + Olaparib	ATM + Olaparib	Radiotherapy	HNSCC cell lines

ATR: ataxia telangiectasia and Rad3-related; ATM: ataxia telangiectasia mutated; DNA-PK: DNA-dependent protein Kinase; HNSCC: head and neck squamous cell carcinoma; HPV(−): HPV-negative; HPV(+): HPV-positive; mTOR: mammalian target of rapamycin; PARP: Poly (ADP-ribose) Polymerase.

## Data Availability

Not applicable.
